# HOSPICE NURSES’ PERSPECTIVES AND CHALLENGES ON HOLISTIC CARE IN MULTIPLE INSTITUTIONS

**DOI:** 10.1093/geroni/igae098.2498

**Published:** 2024-12-31

**Authors:** Ying-Chun Li, Chian-Huei Shiu, Wang-Chuan Juang, Yu-Qi Cheng

**Affiliations:** National Sun Yat-sen University, Kaohsiung, Taiwan (Republic of China); National Sun Yat-sen University, Kaohsiung, Taiwan (Republic of China); Kaohsiung Veterans General Hospital, Kaohsiung Veterans General Hospital, Kaohsiung, Taiwan (Republic of China); National Sun Yat-sen University, Kaohsiung, Taiwan (Republic of China)

## Abstract

Holistic care is a comprehensive care model, including the body, mind, spirit, and society. Palliative care is part of holistic care to improve patient’s quality of life and reduce medical care overuse. Nurses are the primary group providing holistic care. With the increasing care burden of older adults and non-communicable diseases, the demand for palliative care has grown significantly. Taiwan has a growing emphasis on palliative care, and international studies have shown that nurses’ understanding and implementation of holistic care are relatively deficient. Therefore, this study aims to understand hospice nurses’ perceptions and behaviors of holistic care and the challenges or difficulties encountered in its implementation. The study was divided into questionnaires and qualitative interviews based on the knowledge, attitude, and behavior theory. The study samples were from a southern medical center and its collaborative multi-institutions. The questionnaire results indicated that knowledge, attitude, and behavior mutually influence each other, with attitude acting as a complete mediator. Additionally, receiving educational training has a significantly positive impact on both attitude and behavior (p< 0.01). Interview results showed the respondents’ perspectives and behaviors were quite similar to each other. Furthermore, the foremost challenge identified was related to dealing with the patient’s family members. In conclusion, through research to understand hospice nurses’ perceptions and behaviors on holistic care, find out the difficulties and challenges encountered in implementation, and establish appropriate education training and management systems accordingly, which will encourage nurses to provide better holistic care and improve the quality of care.

